# Cardio-Reno-Microvascular Phenotypes and Multifactorial Cardiometabolic Target Achievement in Type 2 Diabetes

**DOI:** 10.3390/jcm15041674

**Published:** 2026-02-23

**Authors:** Silvia Ana Luca, Raluca Malina Bungau, Andreea Herascu, Alin Albai, Sandra Lazar, Bogdan Timar

**Affiliations:** 1 Department of Cardiology, “Victor Babes” University of Medicine and Pharmacy, 300041 Timisoara, Romania; silvia.luca@umft.ro; 2 Centre for Molecular Research in Nephrology and Vascular Diseases, “Victor Babes” University of Medicine and Pharmacy, 300041 Timisoara, Romania; andreea.herascu@umft.ro (A.H.); bogdan.timar@umft.ro (B.T.); 3 Department of Diabetes, “Pius Brinzeu” Emergency Hospital, 300736 Timisoara, Romania; malina.bungau@umft.ro; 4 Second Department of Internal Medicine, “Victor Babes” University of Medicine and Pharmacy, 300041 Timisoara, Romania; 5 First Department of Internal Medicine, “Victor Babes” University of Medicine and Pharmacy, 300041 Timisoara, Romania; sandra.lazar@umft.ro; 6 Department of Hematology, Emergency Municipal Hospital, 300254 Timisoara, Romania

**Keywords:** type 2 diabetes, cardiometabolic targets, phenotypes, cardiovascular diseases

## Abstract

**Background:** Patients with type 2 diabetes (T2D) have high morbidity and mortality rates, mainly due to cardiovascular diseases (CVDs). Given the heterogeneity of this population, in whom atherosclerotic CVD may coexist with varying degrees of microvascular and renal involvement, preventive and therapeutic needs differ among these patients. Multifactorial CV risk factor control has proven beneficial in T2D; however, it remains suboptimal, particularly for lipid and weight targets. **Aims:** The aims were to evaluate, in a real-world cohort of patients with T2D, whether different cardio-reno-microvascular phenotypes are associated with differences in multifactorial cardiometabolic control and to assess individual target attainment along with the use of cardioprotective therapies across phenotypes. **Methods:** In a single-center, cross-sectional study, 174 patients with T2D were enrolled and clustered into four phenotypes based on the presence of atherosclerotic CVD (ASCVD), chronic kidney disease, retinopathy and neuropathy. Achievement of individual and multifactorial cardiometabolic risk factor control was examined across phenotypes. **Results:** More than three quarters of the cohort had ASCVD, microvascular/renal disease, or both. While approximately half of the patients had optimal glycemic control, achievement of LDLC and normal BMI was modest. Target attainment did not differ significantly across phenotypes, with most patients achieving one or two targets and less than one third achieving three or more. Statin use was significantly higher in phenotypes with ASCVD, whereas use of other lipid-lowering therapies remained low. Use of SGLT2is and GLP-1 RAs was also limited. Higher BMI was independently associated with lower odds of multifactorial control. **Conclusions:** In this real-world cohort of patients with T2D, individual and multifactorial cardiometabolic risk factor control was suboptimal, particularly for LDLC and body weight. A phenotype-based approach may help clinicians identify vulnerable subgroups requiring more intensive, risk-based preventive strategies.

## 1. Introduction

### 1.1. Background

Type 2 diabetes (T2D) is a growing public health issue, affecting an increasing proportion of the global population [1]. The high morbidity and mortality rates observed in these patients are closely related to the development and progression of micro- and macrovascular complications, particularly atherosclerotic CVD (ASCVD) [2]. These complications are driven by both the underlying pathophysiological mechanisms observed in T2D and the high burden of cardiovascular (CV) risk factors, with evidence indicating that mortality increases with each additional risk factor present [3]. T2D represents a complex vascular disease in which chronic hyperglycemia and insulin resistance increase oxidative stress, promote endothelial disfunction and accelerate atherosclerosis [4]. Beyond its effects on the CV system, chronic hyperglycemia is also associated with an increased risk of microvascular complications, with landmark trials demonstrating the beneficial effect early glycemic control has on the development and progression of microvascular disease [5]. Since CVDs remain the leading cause of morbidity and mortality in patients with T2D [1], individual CV risk, determined by patients’ history of CVD, conventional CV risk factors, and diabetes-related factors like disease control and duration, plays a central role in determining outcomes [6]. In addition, the presence of renal disease and microvascular complications, including neuropathy and retinopathy, further increase CV risk and clinical frailty [2]. Within the heterogeneous population of patients with T2D, where various patterns of macrovascular, particularly ASCVD, and microvascular complications may coexist, management strategies should be tailored to account for the different preventive measures and levels of therapeutic intensity needed [7].

A multifactorial risk factor control strategy that focuses on simultaneously attaining the recommended targets for hemoglobin A1c (HbA1c), blood pressure (BP), LDL cholesterol (LDLc) and triglycerides (TGs) is associated with a lower risk of CV events, both in patients with and without cardio-renal disease and T2D [3]. However, in a real-world clinical setting, these targets are not optimally achieved [8]. Since most patients with T2D have a very high CV risk and this risk is frequently underestimated, particularly in individuals without established ASCVD [9], clinicians need a simple yet reliable instrument for everyday clinical practice to better identify patients who may benefit from more intensive management strategies. A potentially useful approach is through a complication-based phenotyping strategy that uses a simple clustering method to assess the presence or absence of ASCVD and microvascular/renal disease. This method may help to easily identify more vulnerable, high-risk patients, guide the intensification of BP, lipid, weigh or glycemic management, and increase the use of cardioprotective therapies such as statins, sodium–glucose cotransporter-2 inhibitors (SGLT2is) and glucagon-like peptide-1 receptor agonists (GLP-1 RAs) [10]. However, data on multifactorial cardiometabolic risk factor control across cardio-reno-microvascular phenotypes in patients with T2D in real-world clinical practice remain limited.

### 1.2. Aims

The primary aim of our study was to evaluate whether cardio-reno-microvascular phenotypes, defined based on the presence of ASCVD, renal and microvascular complications, are associated with differences in the attainment of multifactorial CV risk factor control (achievement of ≥greater than or equal to (≥) three of five cardiometabolic risk factors) in a real-world cohort of patients with T2D. To ensure a more practical approach for optimal CV risk factor control in this vulnerable population, secondary aims focused on comparing individual risk factor target achievement and use of cardioprotective therapies across phenotypes. First, we aimed to describe the clinical and laboratory characteristics of each phenotype, including diabetes duration, renal function, prevalence of microvascular disease, blood pressure and lipid parameters. Second, we compared phenotypes in terms of individual CV risk factor target achievement (HbA1c, BP, BMI, TG and LDLC targets defined by the ESC 2023 guidelines) and use of lipid-lowering and cardioprotective therapies. Finally, we examined the association between phenotype clustering and multifactorial CV risk factor control using multivariate analysis adjusted for relevant clinical factors (age, sex, diabetes duration, and BMI). Overall, this simple phenotype-based approach may serve as a practical tool to identify patients who may benefit from more intensive, integrated preventive measures regarding lipid, BP, weight or glycemic control, as well as more systematic use of cardioprotective therapies.

## 2. Materials and Methods

### 2.1. Study Design and Patients

In a single-center, observational, cross-sectional study, 174 adults (50% women) were enrolled between June 2023 and May 2024 according to a consecutive-case principle. Participants were included if the variables required for phenotype construction (history of CVD and indicators of microvascular/renal involvement) and for cardiometabolic target assessment were available from the same index clinical assessment. Patients with type 1 diabetes were excluded. For participants with multiple encounters, the index assessment was defined as the first encounter with complete data for phenotype definition and the primary outcome.

**Figure 1:**
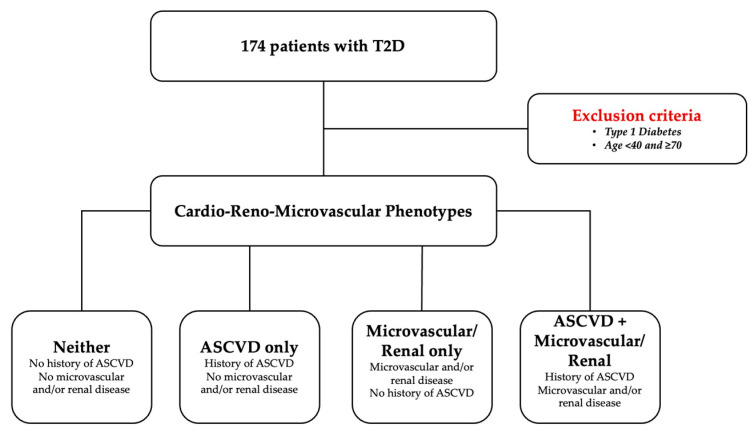
STROBE diagram of the study design.

The study design was constructed to examine phenotype-specific differences in cardiometabolic target achievement and the presence of cardioprotective therapy, using a pragmatic classification integrating documented CVD and microvascular/renal involvement. The STROBE diagram is presented in Figure 1.

The study protocol was approved by the Local Ethics Committee for Scientific Research of “Pius Brînzeu” Emergency Hospital Timisoara (approval number 418/1 November 2023). Data were analyzed in an anonymized format, in accordance with applicable regulations.

### 2.2. Data Collection

Data was extracted from medical records and the institutional electronic database. All measurements were performed during the hospitalization period or at the outpatient visit (index clinical assessment). Collected variables included demographics (age, sex), diabetes duration, anthropometrics (weight, height, BMI), blood pressure, laboratory measures (HbA1c, LDLC, triglycerides, serum creatinine/eGFR, and urinary albumin-to-creatinine ratio), documented diabetic complications (neuropathy, retinopathy), a history of cardiovascular disease, and active cardiometabolic medications at the time of the index visit. All patients had complete data required for phenotype construction. Blood pressure was measured using standardized office methods. Lipid profile (LDL-c, TGs) was determined under fasting conditions. Medication classes of interest were statins, ezetimibe, SGLT2is, and GLP-1 RAs, recorded as binary variables (current use vs. no current use). Given the cross-sectional design of the study, medication adherence could not be independently assessed.

### 2.3. Definitions

History of CVD (ASCVD proxy) was defined based on documented clinical diagnosis in the medical record, including coronary artery disease (prior myocardial infarction/acute coronary syndrome or chronic coronary syndrome), ischemic stroke/transient ischemic attack, peripheral artery disease, and/or prior coronary or peripheral revascularization.

Microvascular involvement included documented diabetic neuropathy and/or diabetic retinopathy. Diabetic neuropathy was defined as loss of peripheral sensitivity on sensory testing. Retinopathy was defined as retinal microvascular changes documented on ophthalmological examination. All data about the presence of microvascular complications were extracted from the patients’ medical records.

Renal involvement was defined as either reduced kidney function or increased albumin excretion. Chronic kidney disease (CKD) was defined as an estimated glomerular filtration rate (eGFR) <60 mL/min/1.73 m^2^ and albuminuria as a urine albumin–creatinine ratio (UACR) ≥30 mg/g. A composite microvascular/renal involvement variable was defined as the presence of at least one of the following: neuropathy, retinopathy, CKD, or albuminuria. eGFR and UACR were extracted from the closest available measurement to the index encounter when multiple results were available.

### 2.4. Phenotype Construction

Participants were categorized into four mutually exclusive phenotypes based on the combination of the ASCVD proxy and microvascular/renal involvement:

- Neither: There was no documented CVD and no microvascular/renal involvement.
- Micro/renal only: There was microvascular/renal involvement without documented CVD.
- ASCVD only: There was documented CVD without microvascular/renal involvement.
- ASCVD + micro/renal: There was documented CVD plus microvascular/renal involvement.

This pragmatic phenotyping approach was selected to capture clinically meaningful strata that may require different intensities of preventive care and facilitate translation into routine practice.

### 2.5. Definition of Cardiometabolic Targets and Outcomes

Cardiometabolic targets were defined as binary indicators of achievement at the index assessment using the following guideline-aligned thresholds:

- HbA1c: <7.0% (53 mmol/mol);
- Body weight: BMI < 25.0 kg/m^2^;
- Blood pressure: systolic BP < 130 mm Hg and diastolic BP < 80 mm Hg;
- Triglycerides: <150 mg/dL (1.7 mmol/L);
- LDLC, risk-specific targets, as follows: moderate risk < 100 mg/dL, high risk < 70 mg/dL, and very high risk < 55 mg/dL (based on CV risk categories as per ESC 2023).

For each participant, the number of achieved targets was counted (0–5). The primary outcome for phenotype analyses was multifactorial control defined a priori as achievement of at least three of the five targets (≥3/5). Secondary descriptive outcomes included phenotype-specific attainment of each target and the distribution of 0–5 achieved targets.

### 2.6. Statistical Analysis

Data was collected using MedCalc Statistical Software version 23.0.8 (MedCalc Software Ltd., Ostend, Belgium) and analyzed using both MedCalc statistical software as well as Python software version 3.13 with the statsmodels package. No formal sample size calculation was performed given the exploratory, real-world nature of the study.

Phenotype distribution is presented as counts and relative frequencies. Continuous variables with a Gaussian distribution are presented as the mean ± standard deviation, while continuous variables with a non-Gaussian distribution are presented as the median and interquartile range. The distribution of the variables was evaluated using Shapiro and Wilk’s method. Baseline characteristics, target achievement, and therapy use were compared across phenotypes using chi-square or Fisher’s exact tests for categorical variables and ANOVA or non-parametric tests (Kruskal–Wallis) for continuous variables, as appropriate.

To assess independent associations between phenotype and multifactorial control (≥3/5 targets), multivariable logistic regression models were fitted with phenotype as the primary predictor (reference: neither), adjusting a priori for age, sex, diabetes duration, and BMI. Results are reported as adjusted odds ratios (ORs) with 95% confidence intervals. Multicollinearity among regression covariates was assessed using variance inflation factors (VIFs) and the condition number, considering VIFs > 5 as suggestive of problematic collinearity. Model performance was evaluated by discrimination (area under the receiver operating characteristic curve, AUC), calibration (Hosmer–Lemeshow goodness-of-fit test) and overall accuracy (Brier score), alongside Nagelkerke’s pseudo-R^2^. Because BMI is also a component of the composite outcome through the normal-weight target, a prespecified sensitivity analysis repeated the multivariable model using multifactorial control defined without the BMI target (≥3 of 4 targets: HbA1c, blood pressure, LDL-C and triglycerides). In addition, we repeated the primary multivariable model excluding BMI from the covariate set to further minimize circularity and confirm the robustness of the phenotype estimates.

In this study, a two-sided *p*-value < 0.05 was considered the threshold for statistical significance.

## 3. Results

### 3.1. Phenotype Distribution

Across the 174 participants, the phenotype distribution was as follows (Table 1): neither, 33 (19.0%); microvascular/renal only, 71 (40.8%); ASCVD only, four (2.3%); and ASCVD + microvascular/renal, 66 (37.9%). Thus, more than three quarters of the cohort exhibited either microvascular/renal involvement, established ASCVD, or both, highlighting a substantial burden of advanced diabetes-related vascular disease.

The ASCVD-only phenotype was uncommon (*n* = 4), a finding consistent with the frequent overlap between macrovascular disease and microvascular/renal complications in long-standing T2D.

### 3.2. Comparison of Patients’ Characteristics by Phenotype

The baseline clinical and laboratory characteristics showed a clear gradient consistent with the intended phenotype definitions. Diabetes duration differed significantly across phenotypes (Kruskal–Wallis *p* < 0.001), being shortest in the neither and ASCVD-only phenotypes and longest in the combined ASCVD + microvascular/renal phenotype. Renal function also differed across phenotypes, with lower eGFR and higher prevalence of CKD in the combined phenotype (eGFR Kruskal–Wallis *p* = 0.003; CKD χ^2^ *p* = 0.002).

Lipid parameters varied across phenotypes. LDLC differed significantly between groups (Kruskal–Wallis *p* = 0.001) and was lowest in the combined ASCVD + micro/renal phenotype, likely reflecting greater use of lipid-lowering therapy in secondary prevention. Triglycerides also showed between-phenotype differences (Kruskal–Wallis *p* = 0.075).

As expected, the prevalence of microvascular disease was concentrated in phenotypes defined by microvascular/renal involvement, with neuropathy and retinopathy markedly more common in the microvascular/renal-only and ASCVD + microvascular/renal groups (neuropathy *p* < 0.001; retinopathy *p* < 0.001). A detailed comparison of parameters between phenotypes is presented in Table 2.

### 3.3. Achievement of Individual Cardiometabolic Targets

Overall achievement of individual targets was heterogeneous. Approximately half of the participants met the HbA1c target, while LDLC target attainment was particularly low. Normal weight (BMI < 25 kg/m^2^) was uncommon, consistent with the high prevalence of obesity observed in the cohort.

Across phenotypes, target attainment did not differ significantly for any of the five targets (HbA1c at target *p* = 0.799; LDLC at target *p* = 0.123; triglycerides at target *p* = 0.255; blood pressure at target *p* = 0.828; normal weight *p* = 0.643). HbA1c control was similar across groups (~45–49% in the neither, micro/renal-only and ASCVD + micro/renal phenotypes), whereas LDLC target attainment remained low in all phenotypes and was numerically highest in the ASCVD + micro/renal phenotype, likely reflecting more intensive lipid-lowering therapy in secondary prevention. Triglyceride target attainment was numerically higher in the neither phenotype, while normal weight (BMI < 25 kg/m^2^) was uncommon across all phenotypes.

Blood pressure control remained suboptimal overall (42.0% at target) and was broadly similar across phenotypes, supporting the need for more effective hypertension management as part of integrated CV prevention. The achievement of individual cardiometabolic targets is presented in Table 3.

### 3.4. Multifactorial Control in T2D

Most participants achieved only one or two targets, and fewer than one third achieved three or more. The proportion of patients achieving ≥3/5 targets was different across phenotypes, although the overall association did not reach statistical significance, likely due to limited power in smaller subgroups (*p* = 0.158).

**Figure 2:**
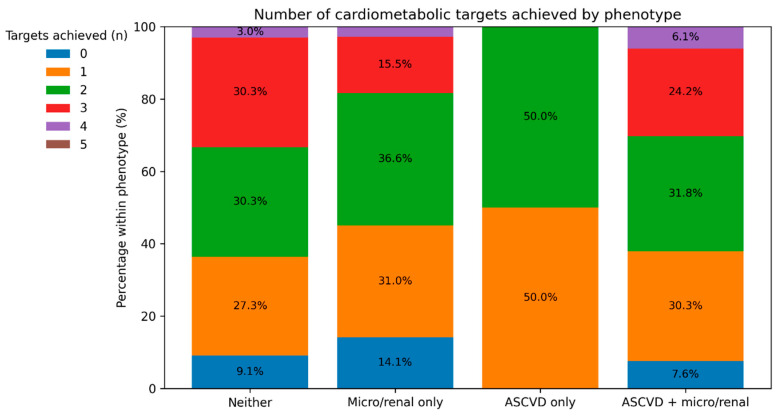
Proportion of number of targets achieved by phenotype.

The distribution of targets achieved (Figure 2) further illustrates the broad opportunity to improve multifactorial care. Notably, approximately one in ten participants achieved none of the five targets, representing a subgroup at especially high preventable risk.

### 3.5. Use of Cardioprotective Therapies by Phenotype

Cardioprotective therapy use differed across phenotypes (Table 4). Statin use was significantly higher in phenotypes with ASCVD, particularly in the combined ASCVD + microvascular/renal group (*p* = 0.002). This aligns with secondary prevention practice and likely contributes to the lower mean LDLC observed in this phenotype.

Despite high-risk profiles, ezetimibe use was low overall, suggesting limited intensification of lipid-lowering therapy beyond statins. No patient used proprotein convertase subtilisin/kexine type 9 inhibitors (PCSK9is). Similarly, uptake of SGLT2is and GLP-1 RAs was modest across phenotypes, indicating room to expand the use of therapies with proven cardiovascular and renal benefits, especially among those with microvascular/renal involvement and established ASCVD.

### 3.6. Multivariable Analysis

In multivariable logistic regression adjusted for age, sex, diabetes duration, and BMI, phenotype category was examined as an independent correlate of achieving ≥3/5 targets (Table 5). Phenotype was not significantly associated with multifactorial control in the adjusted model. In contrast, higher BMI was independently associated with lower odds of multifactorial control, reinforcing excess adiposity as a key barrier to achieving combined cardiometabolic targets.

## 4. Discussion

### 4.1. Interpretation of Findings

The distribution of cardio-reno-microvascular phenotypes in our cohort indicates a high burden of advanced disease: 75% of patients had microvascular/renal disease, ASCVD or both, whereas only a small proportion were classified in the ASCVD-only phenotype, highlighting the frequent overlap of micro- and macrovascular disease in patients with long-standing T2D. A clear clinical gradient was observed across the spectra of the four defined phenotypes. Diabetes duration was longest in the mixed ASCVD and renal/microvascular phenotype, reflecting the high burden of complications observed in long-standing T2D. Renal impairment was most prevalent in the mixed phenotype.

In terms of CV risk factor control, glycemic control was comparable across phenotypes, with approximately half of the patients achieving a HbA1c value below 7%. Although LDLC target attainment was numerically higher in the mixed phenotype, overall achievement was modest, with only about 20% of the cohort reaching the recommended LDLC based on their CV risk category. Normal weight (BMI < 25 kg/m2) was present in only 11% of patients, indicating a high prevalence of obesity in this cohort. Despite examining a cohort of high-risk patients with suboptimal risk factor control, cardioprotective medication use was limited.

Notably, HbA1c and BMI did not differ significantly between phenotypes (Table 2), and HbA1c and weight target attainment were also broadly similar across groups (Table 3). These null associations should not be interpreted causally. By design, our phenotypes reflect the accumulated vascular burden (i.e., the presence of established ASCVD and/or microvascular/renal disease), whereas HbA1c and BMI are contemporaneous, single-timepoint measures that may be substantially influenced by recent clinical management and disease course. In particular, patients who develop microvascular complications may have had prolonged periods of suboptimal glycemic control earlier in the disease trajectory, followed by subsequent treatment intensification once complications were recognized, resulting in HbA1c levels at the index visit that are comparable to those observed in patients without documented complications. Similarly, BMI at a single visit may not capture lifetime exposure to adiposity: weight can decrease over time due to advanced disease, lifestyle interventions, or initiation of weight-modifying therapies, which may reduce between-phenotype contrasts in cross-sectional analyses.

Therefore, the absence of phenotype-related differences in current HbA1c and BMI (Table 2), as well as the lack of phenotype-level associations with individual target achievement (Table 3), likely reflects a combination of reverse causation, therapeutic modification after event/complication onset, and temporal discordance between historical exposures and cross-sectional measurements. These considerations reinforce that our findings primarily describe the real-world distribution of risk factor control and treatment patterns across clinically relevant phenotypes, rather than establishing determinants of complication development. Longitudinal studies incorporating repeated HbA1c and weight trajectories, together with treatment sequencing, are needed to clarify temporality and causal pathways.

From a clinical perspective, the lack of significant differences in terms of CV risk factor control across phenotypes may reflect the overall high burden of risk factors observed in this population, together with the insufficient intensification of strategies targeting weight, BP and lipid management to reduce the onset, recurrence or worsening of diabetes-related complications in patients with T2D.

### 4.2. Strengths and Limitations

This analysis has several strengths. First, the inclusion of a real-world cohort of patients with T2D enrolled in a consecutive-case manner ensures that our findings reflect routine clinical practice. Moreover, clustering patients with T2D into phenotypes based on the presence of microvascular, renal and CV disease represents a pragmatic and reproductible method for use in everyday clinical practice. This strategy facilitates the simultaneous evaluation of cardiometabolic target attainment, distribution of the numbers of targets achieved and use of cardioprotective therapy during a single patient visit. Additionally, as currently recommended by the ESC 2023 guidelines for patients with diabetes, LDLC targets were defined according to the individual CV risk categories.

Several limitations should also be acknowledged. The cross-sectional design of the study cannot establish causality and associations with CV events or prognostic outcomes. Since this was a single-center study conducted in Romania, the generalizability of our findings is limited. The small number of patients classified in the ASCVD-only phenotype may result in imprecise estimations and reduced statistical power to detect real differences across phenotypes. Finally, because BMI is conceptually linked to multifactorial control and the normal-weight criterion is one of the components of the ≥3/5 composite outcome, including BMI as a covariate in the primary regression may raise concerns of circularity. We therefore performed prespecified sensitivity analyses both excluding the BMI target from the composite outcome (≥3/4) and excluding BMI from the covariate set; these analyses yielded consistent phenotype estimates and supported the robustness of our conclusions.

Importantly, the present results are based on individuals with complete data for phenotype construction and target assessment (complete-case analysis). This requirement may have introduced selection bias if patients who were less extensively evaluated were more likely to have missing phenotyping variables and therefore to be excluded. In such a scenario, the prevalence of microvascular/renal disease and ASCVD observed in our analytic sample could over-estimate the true burden in the source population. Accordingly, our estimates should be interpreted as applicable to the subset of patients with available detailed phenotyping, and future studies using strategies to address missingness are warranted to confirm generalizability.

### 4.3. Differences in Relation to Other Studies

Our study examined multifactorial CV risk factor control across four distinct cardio-reno-microvascular phenotypes. To our knowledge, this is one of the first cross-sectional analyses conducted in a European population that compares risk factor control across complication-defined phenotypes in patients with T2D. A study conducted in Japan evaluated risk factor control in patients with and without diabetes-related complications followed in a primary care setting. Although a different phenotypic characterization was used, this study also assessed multifactorial CV risk factor control in patients with varying types of microvascular involvement and CVD. The incidence of microvascular disease was around 28% for each complication; 6.4% patients had microvascular disease affecting three distinct sites (neuropathy, retinopathy, nephropathy), and only 12.6% had CVD. Regarding CV risk factor attainment, about 20% of patients achieved all three targets for HbA1c, BP and lipid control and 11% achieved none; however, the target for LDLC was <120 mg/dL, irrespective of the patient’s baseline CV risk. Patients with better risk factor control had lower rates of microvascular complications, with approximately half of those that achieved all three targets being free of these complications, a proportion that declined with fewer targets achieved. Consistent with our findings, increasing BMI was associated with lower rates of risk factor control and an increased prevalence of retinopathy and nephropathy [11]. The differences in CV risk factor control observed between the previous study and our analysis may, to some extent, be attributed to the inclusion of patients hospitalized for metabolic imbalances in our cohort.

Another study focused on investigating risk factor control across CKD phenotypes, defined based on eGFR levels and albuminuria. Although not specifically investigating risk factor control, the results indicated a higher rate of CV events among patients with CKD and lower eGFR and albuminuria, but not for the phenotype with albuminuria (increased UACR) alone and normal eGFR, highlighting a patient population that may need a more aggressive strategy and encouraging CV risk stratification through a phenotype-based CKD algorithm in patients with T2D. Regarding prescription rates of cardio- and renoprotective therapies, the analysis found that, among patients with diabetic CKD, use of SGLT2is and renin–angiotensin–aldosterone system inhibitors was lower than in the non-CKD group [12].

Additional insights into CV risk factor control in patients with T2D in secondary prevention come from a secondary analysis of three CV outcome trials (CVOTs) conducted by Balasubramanian et al. Patients were clustered into three groups based on their history of events: coronary artery disease (CAD), stroke, both conditions, or none. Risk factor control was considered optimal if at least three factors were within targets (dyslipidemia, BP, smoking status, antithrombotic therapy use). Overall control rates were high, above 80% across all three trials; however, patients with a history of stroke had poorer risk factor control than those with CAD [13]. The high rates of CV risk factor control observed in this analysis may be potentially explained by the definition criteria applied: dyslipidemia was defined as an LDLC < 100 mg/dL or statin use and BP control as values < 140/90 mm Hg. These thresholds are significantly different from current guideline recommendations, which endorse more stringent targets in patients with T2D and established ASCVD: LDLC < 55 mg/dL and BP < 130/80 mm Hg [14]. However, these findings underscore the heterogeneity in risk factor control across complication phenotypes in patients with T2D.

Data from an analysis conducted in the United Kingdom shows that overall achievement of risk factor targets remains low among patients with T2D, with only 6% achieving optimal control of all five assessed risk factors (total cholesterol, TGs, HbA1c, systolic BP, and smoking status). Interestingly, patients with T2D without cardio-renal disease, who were younger and considered to have a lower CV risk, had poorer risk factor control and were less frequently prescribed lipid-lowering and antihypertensive therapies, potentially due to clinical inertia and prescription bias in apparently “healthier” individuals with T2D. However, as reported by Wright et al., this patient subgroup may benefit the most from intensive multifactorial risk factor control [15]. This observation is further supported by data from a Swedish cohort, in which younger individuals with T2D were shown to have the greatest benefits from optimal risk factor control, reinforcing the positive effects of early, intensive risk factor-based strategies in T2D [16]. Although patients without cardio-renal disease may seem lower risk, their baseline CV risk may be substantially underestimated, especially when considering the high risk factor burden, additional microvascular complications, or longer disease duration [17]. Even in the presence of advanced vascular disease, in patients with T2D, multifactorial risk factor control remains beneficial, with better control being strongly associated with lower mortality in those with cardio-renal disease [3].

In our analysis, multifactorial cardiometabolic risk factor control (≥3/5 targets achieved) remained suboptimal across all phenotypes, particularly for LDLC and body weight. A trend towards better control was observed in the mixed ASCVD and microvascular/renal phenotype, although not reaching statistical significance. Consistent with our findings, a recently published large US cohort analysis also reported suboptimal composite risk factor control, with about a third of individuals with T2D achieving ≥three targets. However, patients with ASCVD had improved risk factor control (i.e., ≥three targets) compared to those without, suggesting better preventive strategies in higher risk subgroups. LDLC and BMI were the most challenging to control [18].

Statin use was higher in ASCVD phenotypes, likely reflecting intensified secondary prevention strategies; however, LDLC targets were still modestly achieved, indicating a need to increase the use of nonstatin lipid-lowering therapies like ezetimibe and PCSK9 inhibitors in patients not meeting recommended targets. Our findings are consistent with prior reports from the DAVINCI study regarding LDLC control and lipid-lowering therapy implementation in Central and Eastern Europe. Although not limited to patients with T2D, LDLC target attainment was below 25% overall, with even lower rates in secondary prevention. In Romania, approximately one third of the patients included in the study met the 2019 ESC guideline-recommended targets for LDLC, and although this was higher than in our cohort, it indicates that additional barriers in the optimal implementation of lipid-lowering strategies in patients with T2D are still present. It should be noted that management strategies regarding CV risk assessment and lipid targets used in the Romanian population are aligned with the recommendations endorsed by the European Society of Cardiology. Use of combination lipid-lowering therapy with ezetimibe and PCSK9 inhibitors was exceptionally low (5% and zero, respectively) [19].

More ambitious lipid targets are currently recommended by European guidelines. The 2023 guideline edition imposes an LDLC target of <55 mg/dL in patients with T2D at very high CV risk [14]. Since in real-world practice approximately half of these patients fall into the very high CV risk category, most patients with T2D treated in routine clinical practice remain far from achieving these targets [9]. The newly published 2025 ESC guideline on dyslipidemias maintains the same LDLC targets for the moderate-, high-, and very high-risk categories, including for patients with T2D, but introduces a novel extreme-risk category. This category includes patients with recurrent vascular events despite maximal statin therapy or with vascular disease involving multiple arterial territories. In this subgroup, an LDLC of <40 mg/dL is considered the desired target for lipid control [20].

Beyond lipid control, optimal weight management is another key factor in mitigating CV risk. Higher BMI is associated with increased morbidity and mortality, as well as lower rates of CV risk factor control, reinforcing the importance of managing obesity in this population [21]. Novel cardioprotective agents, like SGLT2is and GLP-1 RAs, are currently recommended as first-line therapies in patients with T2D and cardiovascular and/or renal disease [14]. In addition to their glucose-lowering properties, these agents also influence multiple CV risk factors, particularly BP and body weight [22]. GLP-1 RAs have an established role in the pharmacological management of obesity, both in subjects with and without T2D [23]. SGLT2is exert complementary hemodynamic effects, particularly beneficial in heart failure and CKD patients, along with other pleiotropic effects like reducing inflammation and oxidative stress [24]. Also, by decreasing sympathetic activity, these agents have been shown to reduce the incidence of arrhythmias and the risk of sudden cardiac death [25]. Despite this, prescription rates of these therapies remain low, particularly in high-risk subgroups, such as patients with ASCVD or CKD [26,27]. These findings call for a multidisciplinary approach with improved risk stratification and more intensive, risk factor-based treatment strategies to reduce CV risk in patients with T2D.

In our multivariable model, BMI emerged as the only independent correlation of composite target non-achievement. Importantly, collinearity between covariates was unlikely to explain this finding, as multicollinearity diagnostics showed low variance inflation factors (all VIFs ≤ 2.05) and a low condition number. A clinically plausible interpretation is that excess adiposity represents a central upstream driver that simultaneously impairs several cardiometabolic domains (blood pressure, triglycerides and glycemic control) and reflects a long-term lifestyle and metabolic burden. In addition, because the normal-weight criterion (BMI < 25 kg/m^2^) is one of the components of the composite endpoint, BMI is intrinsically linked to the probability of reaching ≥3 targets. Consistently, when we repeated the analysis using a sensitivity definition of multifactorial control that excluded the BMI component (≥3 of 4 targets: HbA1c, blood pressure, LDL-C and triglycerides), BMI was no longer independently associated with the outcome. Taken together, these results emphasize that weight management is a key actionable barrier to comprehensive risk factor control in routine T2D care and that composite endpoints including a weight component should be interpreted with this structural relationship in mind.

Potential factors that may also contribute to the low achievement rates of CV risk targets in patients with T2D include the following: First, disparities in healthcare resources related to treatment cost may influence prescription rates of novel glucose-lowering medications [28]. Drug-related side effects, like gastrointestinal events with incretin-based medications [29], may also contribute to the modest uptake of these agents observed in our cohort. True statin intolerance or the nocebo effect could also affect lipid target achievement [30]. However, treatment adherence was not formally addressed in our study.

### 4.4. Relevance of Our Findings

Our findings introduce a simple, phenotype-based algorithm suitable for everyday clinical practice, which may facilitate the systematic screening of patients with T2D at every visit and help identify those at high risk. These include individuals with long diabetes duration and multiple vascular complications or comorbidities, those with CKD or albuminuria and very high CV risk, or patients that need more intensified prevention strategies and more frequent follow-ups. This strategy could also support clinical care organization and provide a rationale for an integrated, multidisciplinary strategy involving medical specialties like diabetology, cardiology and nephrology and improving the implementation of more intensified protocols for BP, lipid and weight management in patients with T2D.

In terms of health policies, a phenotype-based strategy may encourage clinicians to increase prescription rates of cardioprotective therapies, like SGLT2is and GLP-1 RAs, while also prompting a more aggressive lipid control strategy through improved statin use and nonstatin add-on therapies.

As a key practical message, our analysis highlights the persistent lack of optimal CV risk factor control among very high-risk subgroups, like individuals with both ASCVD and microvascular and/or renal involvement. Phenotyping patients with T2D should therefore serve as a trigger for action, not just as a classification tool, ultimately improving outcomes in the particularly vulnerable population.

## 5. Conclusions

In this real-world cohort of adults with T2D, cardiometabolic target attainment was suboptimal, with particularly low achievement of LDLC and body-weight targets and only a minority of patients reaching multifactorial control (≥3 of 5 targets). A pragmatic phenotyping approach based on documented ASCVD and microvascular/renal involvement revealed a high burden of advanced vascular disease, with most patients presenting either microvascular/renal complications, established cardiovascular disease, or both. While treatment patterns, like higher statin use in ASCVD phenotypes, partially reflected risk, important therapeutic gaps persisted, including limited intensification of lipid-lowering therapy and modest uptake of cardio-renal-protective glucose-lowering agents. Overall, these findings support the clinical value of simple phenotyping as a practical framework to identify vulnerable subgroups and to prompt risk-based intensification of integrated cardiometabolic prevention in routine diabetes care.

## Figures and Tables

**Table 1:** Distribution of phenotypes in T2D.

Phenotype	Patients
Neither	33 (19.0%)
Microvascular/renal only	71 (40.8%)
ASCVD only	4 (2.3%)
ASCVD + microvascular/renal	66 (37.9%)

Results are presented as counts (relative frequencies). Table legend: ASCVD—atherosclerotic cardiovascular disease.

**Table 2:** Patients’ characteristics by phenotype.

Variable	Overall	Neither	Microvascular/Renal Only	ASCVD Only	ASCVD + Microvascular/Renal	*p*-Value
Age (years) ^a^	61.0 [53.2–65.0]	58.0 [52.0–63.0]	61.0 [52.5–65.0]	54.5 [51.5–58.8]	62.0 [58.0–66.0]	0.168
Diabetes duration (years) ^a^	7.0 [2.0–13.8]	3.0 [1.0–8.0]	7.0 [2.0–12.5]	2.5 [0.1–5.2]	10.0 [5.2–16.8]	<0.001
BMI (kg/m^2^) ^b^	31.3 ± 5.7	29.9 ± 4.8	32.3 ± 6.1	31.2 ± 8.6	31.0 ± 5.4	0.239
HbA1c (%) ^a^	7.2 [6.2–8.7]	7.2 [6.5–8.2]	7.1 [6.2–8.6]	9.2 [6.9–11.8]	7.4 [6.2–8.8]	0.825
LDLc (mg/dL) ^a^	89.5 [62.0–128.0]	106.0 [68.0–128.0]	100.0 [71.0–140.0]	128.5 [101.2–142.2]	74.0 [55.0–104.5]	0.001
Triglycerides (mg/dL) ^a^	141.0 [111.2–202.0]	119.0 [97.0–153.0]	144.0 [117.5–226.0]	147.5 [131.5–164.2]	144.5 [108.8–192.5]	0.075
Systolic BP (mmHg) ^a^	130.0 [125.0–140.0]	130.0 [130.0–140.0]	130.0 [130.0–140.0]	125.0 [115.0–131.2]	130.0 [125.0–145.0]	0.471
Diastolic BP (mmHg) ^a^	80.0 [75.0–90.0]	80.0 [80.0–90.0]	80.0 [75.0–90.0]	85.0 [75.0–91.2]	80.0 [75.0–85.0]	0.679
eGFR (mL/min/1.73 m^2^) ^a^	87.0 [72.0–103.8]	98.0 [86.0–106.0]	95.0 [77.0–106.0]	89.0 [86.8–97.2]	83.0 [55.5–99.5]	0.003
UACR (mg/g) ^a^	11.2 [5.3–29.4]	5.7 [4.0–10.7]	14.9 [6.7–47.1]	4.7 [2.7–6.1]	13.5 [7.0–40.0]	<0.001
Female sex ^c^	87 (50.0%)	16 (48.5%)	37 (52.1%)	1 (25.0%)	33 (50.0%)	0.763
eGFR < 60 mL/min ^c^	26 (14.9%)	0 (0.0%)	8 (11.3%)	0 (0.0%)	18 (27.3%)	0.002
Albuminuria ^c^	41 (23.6%)	0 (0.0%)	20 (28.2%)	0 (0.0%)	21 (31.8%)	0.002
Neuropathy ^c^	119 (68.4%)	0 (0.0%)	59 (83.1%)	0 (0.0%)	60 (90.9%)	<0.001
Retinopathy ^c^	41 (23.6%)	0 (0.0%)	16 (22.5%)	0 (0.0%)	25 (37.9%)	<0.001
Obesity (BMI ≥ 30 kg/m^2^) ^c^	103 (59.2%)	16 (48.5%)	44 (62.0%)	2 (50.0%)	41 (62.1%)	0.538

^a^ Continuous variable with non-Gaussian distribution. Results are presented as median [interquartile range]. *p*-value was calculated using Kruskal–Wallis test. ^b^ Continuous variables with Gaussian distribution. Results are presented as mean ± standard deviation. *p*-value was calculated using ANOVA test. ^c^ Nominal variables. Results are presented as counts (relative frequencies). *p*-value was calculated using Fisher’s exact test. Table legend: BMI—body mass index; HbA1c—hemoglobin A1c; LDLc—low-density lipoprotein cholesterol; BP—blood pressure; eGFR—estimated glomerular filtration rate; UACR—urinary albumin-to-creatinine ratio.

**Table 3:** Achievement of individual risk factor targets by phenotype.

Target	Overall	Neither	Micro/Renal Only	ASCVD Only	ASCVD + Micro/Renal	*p*-Value
HbA1c at target	81 (46.6%)	15 (45.5%)	35 (49.3%)	1 (25.0%)	30 (45.5%)	0.799
LDLc at target	35 (20.1%)	6 (18.2%)	10 (14.1%)	0 (0.0%)	19 (28.8%)	0.123
Triglycerides at target	101 (58.0%)	24 (72.7%)	37 (52.1%)	2 (50.0%)	38 (57.6%)	0.255
Blood pressure at target	73 (42.0%)	14 (42.4%)	27 (38.0%)	2 (50.0%)	30 (45.5%)	0.828
Normal weight (BMI < 25)	20 (11.5%)	4 (12.1%)	6 (8.5%)	1 (25.0%)	9 (13.6%)	0.643

Nominal variables. Results are presented as counts (relative frequencies). *p*-value was calculated using Fisher’s exact test. Table legend: HbA1c—hemoglobin A1c; LDLc—low-density lipoprotein cholesterol; BMI—body mass index.

**Table 4:** Use of cardioprotective therapies by phenotype.

Therapy	Overall	Neither	Microvascular/Renal Only	ASCVD Only	ASCVD + Microvascular/Renal	*p*-Value
Statin	113 (64.9%)	16 (48.5%)	40 (56.3%)	3 (75.0%)	54 (81.8%)	0.002
Ezetimibe	14 (8.0%)	1 (3.0%)	4 (5.6%)	0 (0.0%)	9 (13.6%)	0.186
SGLT2 inhibitor	47 (27.0%)	8 (24.2%)	20 (28.2%)	0 (0.0%)	19 (28.8%)	0.623
GLP-1 RA	36 (20.7%)	9 (27.3%)	12 (16.9%)	0 (0.0%)	15 (22.7%)	0.440

**Table 5:** Multivariable logistic regression model for multifactorial control (≥3 of 5 targets) by phenotype.

Predictor	OR (95% CI)	*p*-Value
ASCVD + microvascular/renal vs. neither	1.08 (0.41–2.89)	0.873
Microvascular/renal only vs. neither	0.57 (0.22–1.51)	0.260
Age (per 1-year increase)	0.99 (0.94–1.04)	0.750
Male gender (vs. female)	1.50 (0.72–3.11)	0.282
Diabetes duration (per 1-year increase)	0.97 (0.92–1.03)	0.345
BMI (per 1 kg/m^2^ increase)	0.91 (0.85–0.98)	0.013

## Data Availability

The data presented in this study are available on request from the corresponding author. The data are not publicly available due to the hospital’s privacy policy.

## Abbreviations

The following abbreviations are used in this manuscript:

ASCVD	Atherosclerotic cardiovascular disease
BMI	Body mass index
BP	Blood pressure
CKD	Chronic kidney disease
CV	Cardiovascular
CVDs	Cardiovascular diseases
eGFR	Estimated glomerular filtration rate
ESC	European Society of Cardiology
GLP-1 RAs	Glucagon-like peptide-1 receptor agonists
HbA1c	Hemoglobin A1c
LDLc	Low-density lipoprotein cholesterol
OR	Odds ratio
PCSK9	Proprotein convertase subtilisin/kexin type 9
SGLT2is	Sodium–glucose cotransporter-2 inhibitors
T2D	Type 2 diabetes
TGs	Triglycerides
TOD	Target organ damage
UAC	Urine albumin–creatinine
